# Characterizing the influence of various antimicrobials used for metaphylaxis against bovine respiratory disease on host transcriptome responses

**DOI:** 10.3389/fvets.2023.1272940

**Published:** 2023-10-05

**Authors:** Rebecca A. Bigelow, John T. Richeson, Molly McClurg, Robert Valeris-Chacin, Paul S. Morley, Jenna L. Funk, Matthew A. Scott

**Affiliations:** ^1^Department of Agricultural Sciences, West Texas A&M University, Canyon, TX, United States; ^2^Veterinary, Education, Research, and Outreach Program, School of Veterinary Medicine and Biomedical Sciences, Texas A&M University, Canyon, TX, United States

**Keywords:** bovine respiratory disease, metaphylaxis, antimicrobial, transcriptome, RNA-Seq, immune, cattle, T-cell

## Abstract

Currently, control against bovine respiratory disease (BRD) primarily consists of mass administration of an antimicrobial upon arrival to facility, termed “metaphylaxis.” The objective of this study was to determine the influence of six different antimicrobials used as metaphylaxis on the whole blood host transcriptome in healthy steers upon and following arrival to the feedlot. One hundred and five steers were stratified by arrival body weight (BW = 247 ± 28 kg) and randomly and equally allocated to one of seven treatments: negative control (NC), ceftiofur (CEFT), enrofloxacin (ENRO), florfenicol (FLOR), oxytetracycline (OXYT), tildipirosin (TILD), or tulathromycin (TULA). On day 0, whole blood samples and BW were collected prior to a one-time administration of the assigned antimicrobial. Blood samples were collected again on days 3, 7, 14, 21, and 56. A subset of cattle (*n* = 6) per treatment group were selected randomly for RNA sequencing across all time points. Isolated RNA was sequenced (NovaSeq 6,000; ~35 M paired-end reads/sample), where sequenced reads were processed with ARS-UCD1.3 reference-guided assembly (HISAT2/StringTie2). Differential expression analysis comparing treatment groups to NC was performed with glmmSeq (FDR ≤ 0.05) and edgeR (FDR ≤ 0.1). Functional enrichment was performed with KOBAS-i (FDR ≤ 0.05). When compared only to NC, unique differentially expressed genes (DEGs) found within both edgeR and glmmSeq were identified for CEFT (*n* = 526), ENRO (*n* = 340), FLOR (*n* = 56), OXYT (*n* = 111), TILD (*n* = 3,001), and TULA (*n* = 87). At day 3, CEFT, TILD, and OXYT shared multiple functional enrichment pathways related to T-cell receptor signaling and FcεRI-mediated NF-kappa beta (kB) activation. On day 7, Class I major histocompatibility complex (MHC)-mediated antigen presentation pathways were enriched in ENRO and CEFT groups, and CEFT and FLOR had DEGs that affected IL-17 signaling pathways. There were no shared pathways or Gene Ontology (GO) terms among treatments at day 14, but TULA had 19 pathways and eight GO terms enriched related to NF- κβ activation, and interleukin/interferon signaling. Pathways related to cytokine signaling were enriched by TILD on day 21. Our research demonstrates immunomodulation and potential secondary therapeutic mechanisms induced by antimicrobials commonly used for metaphylaxis, providing insight into the beneficial anti-inflammatory properties antimicrobials possess.

## 1. Introduction

Bovine respiratory disease (BRD) is a multifactorial disease complex that has been identified as the cause of approximately 75% of morbidity and over 50% of mortality in the feedlot industry (1, 2). The pathogenesis of BRD culminates from the interactions between stressors and environmental risk factors, viral and bacterial pathogens, and the host immunological response (3). Due to the structure of the beef cattle marketing system, calves are exposed to multiple stressors at once, which can result in host immunosuppression. A viral infection allows for pathogenic bacteria to induce a secondary infection, contributing to the development of BRD (4). Moreover, current BRD diagnosis in the field is subjective, based on visual assessment of clinical signs associated with BRD, and has previously been shown to have poor sensitivity (5, 6). As such, metaphylaxis, or the mass administration of injectable antimicrobials upon facility arrival, is an effective method of controlling BRD (7). With the rise of antimicrobial resistance (AMR), exploring mechanisms of how drugs influence host immunological responses is imperative to understanding how they remain efficacious (8). Examining the host whole blood transcriptome and differential gene expression provides insight to novel genomic mechanisms associated with immune response to antimicrobial administration.

RNA sequencing (RNA-Seq) produces quantitative and qualitative data regarding RNA in a biologic sample. It accomplishes this by combining high-throughput sequencing methodology with computational analysis to quantify transcripts in an RNA extraction (9). This technology can be used to identify transcribed genes within a cell or tissue, genomic activity at a particular time, and associated mechanisms being up- or downregulated over time. RNA-Seq has been used to examine development of BRD in multiple tissues against different pathogens, differences between sick versus healthy cattle, identification of potential biomarkers for BRD, and antimicrobial resistance genes among various bacteria (10–15). However, RNA-Seq has not been used to specifically investigate the impact of antimicrobial administration on the host immunological response through the whole blood transcriptome.

Therefore, the objective of this study was to evaluate the effect of six different antimicrobials used for metaphylaxis on the host whole blood transcriptome of healthy steers upon and following arrival to the feedlot. The findings of this study may provide a foundation for future research into secondary mechanisms identified as potential therapeutic opportunities for BRD.

## 2. Materials and methods

Animal procedures were approved by the West Texas A&M University (WTAMU) Institutional Animal Care and Use Committee before study initiation (IACUC protocol #2022.03.002). This research was conducted from May 2022 to July 2022 at the WTAMU Research Feedlot, near Canyon, TX. This study was carried out in accordance with Animal Research: Reporting of *In Vivo* Experiments (ARRIVE) guidelines (16).

### 2.1. Arrival processing

A total of 105 crossbred steers (243 ± 23.9 kg) were acquired from a local grow yard facility in the panhandle of Texas. Previous health history records indicated that all cattle received tildipirosin (Zuprevo, Merck Animal Health, Madison, NJ), a trenbolone acetate and estradiol implant (Revalor-G implant, Merck Animal Health, Madison, NJ), an antiparasitic drug (Dectomax, Zoetis, Parsippany-Troy Hills, NJ), an IBR/BRSV/PI_3_ vaccine (Nasalgen, Merck Animal Health, Madison, NJ), Synanthic oral dewormer (Boehringer Ingelheim, Ridgefield, CT), a Titanium 5 vaccine for IBR, BVD Types I and II, PI_3_, and BRSV (Elanco Animal Health, Greenfield, IN), and a Bovilis Vision vaccine (Merck Animal Health, Madison, NJ) at label dosing during the start of their backgrounding period, 45 days prior to arrival at the WTAMU Research Feedlot. Prior to animal arrival and every sample collection day, the squeeze chute scale used in this study was validated by placing 45.54 kg (50-lb certified steel weights) on each side of the chute at a time until a total of 454 kg was validated. Upon arrival (day −1), individual body weight (BW) was recorded. Additionally, cattle were given identification ear tags and were confirmed to be negative for persistent infection with bovine viral diarrhea virus (BVDV) *via* ear notch antigen capture ELISA (PI; Cattle Stats, Oklahoma City, OK). Steers were placed in a single 124.8 m^2^ pen together overnight with *ad libitum* access to water, a starter diet of 0.5% of their arrival BW, and coastal Bermuda grass hay at 0.5% arrival BW.

On day 0, individual BW were collected again and averaged with day −1 BW to determine initial BW. Whole blood samples were collected *via* jugular venipuncture into PAXgene Blood RNA tubes (QIAGEN, Germantown, MD) prior to a one-time administration of the assigned treatment at day 0. Nasopharyngeal swabs and fecal samples were also collected on day 0. Blood, BW, and nasopharyngeal swabs were collected again on days 3, 7, 14, 21, and 56, with fecal samples collected on days 21 and 56; nasopharyngeal swabs and fecal samples were collected for a separate study. Cattle were evaluated daily for bovine respiratory disease (BRD), or other signs of clinical disease, by a trained observer and assigned a clinical illness score for BRD (CIS: 0 to 4 scale). A CIS of 0 is described as a “normal” animal showing no clinical signs of illness. A CIS of 1 indicated “mild BRD” with elevated respiratory rate, mild-to-moderate anorexia, mild depressed attitude, and a shallow, dry cough when viewed at a distance. A CIS of 2 indicated a “moderate BRD” steer with a gaunt appearance, possible nasal and/or ocular discharge, mild-to-moderate muscle weakness and depressed attitude, and a persistent shallow, dry cough. A steer assigned a CIS of 3 was considered “severe BRD” with labored breathing, purulent nasal and/or ocular discharge, productive coughing, loss of alertness, and severe depression. A CIS of 4 was deemed “moribund” that was unresponsive upon human approach, recumbent and unwilling to rise when approached, showed evidence of moderate-to-severe dehydration, and marked anorexia. Any steer deemed a CIS of a 1 or 2 was pulled and treated if the rectal temp recorded was ≥40° C. A steer with a CIS of 3 or 4 was treated regardless of rectal temp recorded. If a steer was assigned a CIS of 4 and deemed unlikely to clinically recover by an on-site veterinarian, it was euthanized *via* AMVA-approved methods. If a steer was pulled but not treated, it was returned to its treatment pen. However, if a steer was pulled and treated, a blood sample, nasopharyngeal swab, and fecal sample were taken before removing the animal from trial. No cattle were pulled nor treated for clinical disease after their initial administration of treatment at any time throughout this 56-day trial.

### 2.2. Treatments and experimental design

The BWs collected on day −1 were used to randomly allocate cattle to experimental treatments with equal weights *via* Excel RANDBETWEEN function (Microsoft, Redmond, WA). Within each treatment pen, there were 15 individuals total. In the pens receiving antibiotics, ten individuals were randomly selected for the administration of antimicrobial treatment and five received no antimicrobial treatment, serving as sentinel controls within each treatment pen. Cattle within pens were randomly selected using a random number generator in Excel, selecting the five lowest values as sentinel controls *via* RANDBETWEEN function. This experiment consisted of seven treatment groups evaluated over a 56-day receiving period: (1) negative control, no antimicrobial administration (**NC**), (2) ceftiofur administered subcutaneously at the base of the ear at a dosage of 6.6 mg CE/kg BW (EXCEDE Sterile Suspension, Zoetis, Parsippany-Troy Hills, NJ) (**CEFT**), (3) enrofloxacin administered subcutaneously in the neck at a dosage of 12.5 mg/kg BW (Baytril 100, Elanco Animal Health, Greenfield, IN) (**ENRO**), (4) florfenicol administered subcutaneously in the neck at a dosage of 40 mg/kg BW (NUFLOR Injectable Solution, Merck Animal Health, Madison, NJ), (5) oxytetracycline administered subcutaneously in the neck at a dosage of 4.1 mg/kg BW (Noromycin 300 LA, Lenexa, KS) (**OXYT**), (6) tildipirosin administered subcutaneously in the neck at a dosage of 6.2 mg/kg BW (Zuprevo, Merck Animal Health, Madison, NJ) (**TILD**), (7) tulathromycin administered subcutaneously in the neck at a dosage of 2.5 mg/kg BW (DRAXXIN Injectable Solution, Zoetis, Parsippany-Troy Hills, NJ) (**TULA**). All treatments were administered one time per label dosing and administration at day 0, following Beef Quality Assurance guidelines. To minimize interference between treatments, an empty pen was left between treatment groups to eliminate nose-to-nose contact. Following treatment allocation and sampling on day 0, the NC cattle were processed first followed by ENRO, FLOR, TULA, TILD, CEFT, and OXYT every sampling day. The sampling order of treatment groups was consistent throughout this trial. The chute, tub, and holding pens were physically cleaned with soap and brushes, then disinfected with Virkon S (Lanxess AG, Cologne, Germany) per label instructions between sampling each treatment group across all timepoints following day 0.

### 2.3. Housing and management

Steers were housed in soil-surfaced pens with 20.8 meters^2^ of space allowance. The linear bunk space per animal was 50.6 centimeters, and the cattle were fed the same starter diet throughout the entire 56-day trial. Cattle were fed once daily at approximately 1,000 h, and feed bunks were visually evaluated twice daily at 0630 and 2,130 h to determine adjustments to feed offering. Feed bunks were managed according to standard operating procedure at the WTAMU Research Feedlot, with a goal of little to no feed remaining at 0630 the following morning. Feed samples were collected twice weekly for dry matter (DM) determination and a diet composite was taken every 2 weeks for nutrient composition analysis at a commercial laboratory (Servi-tech Labs, Amarillo, TX; Supplementary File S1). Orts were collected, weighed, and analyzed for DM determination in a forced-air oven at 40.5° C for 24 h and used to adjust DM intake.

### 2.4. Sample collection and RNA extraction

On days 0, 3, 7, 14, 21, and 56, blood samples were collected into PAXgene RNA Blood Tubes (QIAGEN, Germantown, MD) and placed on ice packs at approximately 4° C for transportation to the Texas A&M University Veterinary Education, Research, and Outreach laboratory (Canyon, TX) where they were frozen at −80° C until RNA extractions were performed. From the total pool of 630 samples, a subset (*n* = 252) was selected for RNA extraction and sequencing. For subset selection, six random animals were chosen from each treatment pen over all six timepoints, with the exclusion of sentinel controls due to budgetary constraints. The PAXgene Blood miRNA Kit (QIAGEN, Germantown, MD) was used in conjunction with automated processing *via* a QIAcube Connect device (QIAGEN, Germantown, MD) to isolate total RNA from all samples according to manufacturer protocol. Following isolation, RNA quantity (mean RNA yield = 2864.9 ± 1319.5 ng) and quality (mean RIN = 8.4 ± 0.7) were measured *via* a Qubit Flex Fluorometer (Thermo Fisher Scientific, Waltham, MA) and TapeStation 4,200 (Agilent Technologies, Santa Clara, CA), respectively. Complete metadata table, including breed identity based on visual inspection (Btau: *Bos taurus*; Ind: *Bos indicus*), for each individual sample is found in Supplementary File S2.

### 2.5. RNA sequencing and bioinformatic processing

Library preparation was performed with the Stranded mRNA Library Kit (Illumina, San Diego, CA) per manufacturer’s instruction, with 150 bp paired-end sequencing (2×150) performed with a NovaSeq 6,000 S4 v1.7+ (Illumina, San Diego, CA; S4 reagent kit, v1.5) across three lanes at the North Texas Genome Center (NTGC, Arlington, TX); sequencing resulted in a median of 34.1 ± 5.6 M paired-end reads per sample. Following sample demultiplexing *via* bcl2fastq2 v2.20, raw sequenced reads were quality assessed with FastQC v0.11.9^1^ and MultiQC v1.12 (17). Reads were subsequently trimmed for ambiguous base calling, retained Illumina adaptors, and minimum read lengths with Trimmomatic v0.39 (18) (mean retainment: 99.486%; σ = 0.003%) using the following parameters: “ILLUMINACLIP:TruSeq3.fa:2:30:10:2:TRUE,” “SLIDINGWINDOW:4:20,” “MINLEN:28,” “LEADING:3,” and “TRAILING:3.” Following read quality assessment and trimming, retained trimmed reads were mapped and indexed to the *Bos taurus* reference assembly ARS-UCD1.3 *via* HISAT2 v2.21 (19) with default parameters. Sequence Alignment Map (.sam) files generated from HISAT2 alignments were converted to Binary Alignment Map (.bam) files *via* Samtools v1.14 (20), with default parameters, prior to transcript assembly. Transcript assembly and relative gene-level expression estimation was performed *via* StringTie2 v2.2.0 (21), with default parameters and workflow described by Pertea and colleagues (22). Following merged Gene Transfer Format (.gtf) file generation of expression estimates for each sample, post-processing for the appending of ambiguous gene-level identifications (“MSTRG” tags) was performed with a custom Perl script provided by Pertea.^2^ Raw gene-level count matrices for each sample were generated with the Python3 script prepDE.py3 (22), selecting for an average read length (“– 1”) of 150 and all other parameters set to default. All raw sequencing data and curated metadata produced by this study are available at the National Center for Biotechnology Information Gene Expression Omnibus (NCBI-GEO) under the accession number GSE225025.

### 2.6. Differential gene expression analysis

Raw gene counts generated for each sample were processed and analyzed in RStudio v2022.02.3 + 492 with the Bioconductor package edgeR v3.40.2 (23, 24). All analyses were performed as treatment groups versus NC (CEFT vs. NC, ENRO vs. NC, etc.) across all six timepoints. The ComBat_seq function in the sva package v3.46.0 was used to adjust for batch effects, in the form of sequencing lanes, using an empirical Bayes framework in the raw gene counts (25); this was applied to all sequencing libraries at the same time. Raw counts were processed and filtered using the filterByExpr function in the edgeR package as described by Chen et al. (26), utilizing gene counts-per-million (CPM) of 0.2 across a minimum of 12 samples. Library normalization was performed with the trimmed means of M-values method (TMM) (27). Differentially expressed genes (DEGs) were identified through pairwise comparison of NC and each treatment group using likelihood ratio testing (glmLRT) where DEGs were considered significant with a false discovery rate (FDR) of ≤0.1. Specifically, all edgeR analyses were performed within each time point (ex. Day 0, Day 3, etc.) between each treatment group versus the negative control group (ex. CEFT vs. NC, ENRO vs. NC, etc.). The package glmmSeq v.0.2.0 was used to investigate changes in gene expression between treatment groups (28). In this generalized linear mixed model, timepoint, group and the interaction of timepoint:group were analyzed as fixed effects with breed and animal ID included as random effects. Heatmapping was performed using the R package pheatmap v1.0.12,^3^ utilizing data-centered and normalized z-scores from log2CPM TMM-normalized gene counts; Ward’s minimum variance method was used with calculated Spearman correlation coefficients, further grouped through k-means clustering, and Minkowski distances for clustering dissimilarities by row (gene) and column (sample), respectively. The list of DEGs found in the glmmSeq and edgeR models were compared in Excel, and DEGs identified through group-level comparisons which were shared between the lists at each timepoint were identified using a conditional formatting rule that highlighted duplicate DEGs between the lists of genes from glmmSeq and edgeR. The matching DEGs were then annotated using the National Center for Biotechnology (NCBI) gene database using the bovine and human reference genomes to annotate “LOCI” genes when required. Functional enrichment was performed on the resulting lists of shared DEGs at each timepoint found between the two analyses (glmmSeq and edgeR).

### 2.7. Downstream analysis of DEGs

Pathway analysis and Gene Ontology (GO) were performed using KOBAS-intelligence v.3.0 (29). Overrepresentation analysis used hypergeometric distribution and Fisher’s exact testing to evaluate whether the gene symbols entered were overrepresented in a specific functional gene set. Pathway databases utilized were KEGG and Reactome (30, 31). Benjamini-Hochberg procedure was used for multiple hypothesis correction, and the FDR cutoff for significance was 0.05 (32). GO terms consist of biological processes, molecular functions, and cellular components that the identified DEGs enriched. Specifically, functional enrichment analyses were conducted in two parts for each treatment group: first by DEGs identified through group-level comparisons (i.e., shared genes between glmmSeq (group) and edgeR glmLRT pairwise testing) and, second, by those DEGs identified by glmmSeq timepoint:group interactions. Additionally, principal component analysis (PCA) was conducted with the Bioconductor package PCAtools v.2.10.0.^4^ Metadata components for PCA correlational analysis included average daily gain (ADG), animal ID, timepoint of sample collection, and treatment group. Intervene was used to compute pairwise intersections and plot-associated metrics as correlation plots for DEGs of each treatment group at every timepoint (33).

## 3. Results

### 3.1. Identification of DEGs

**Figure 1:**
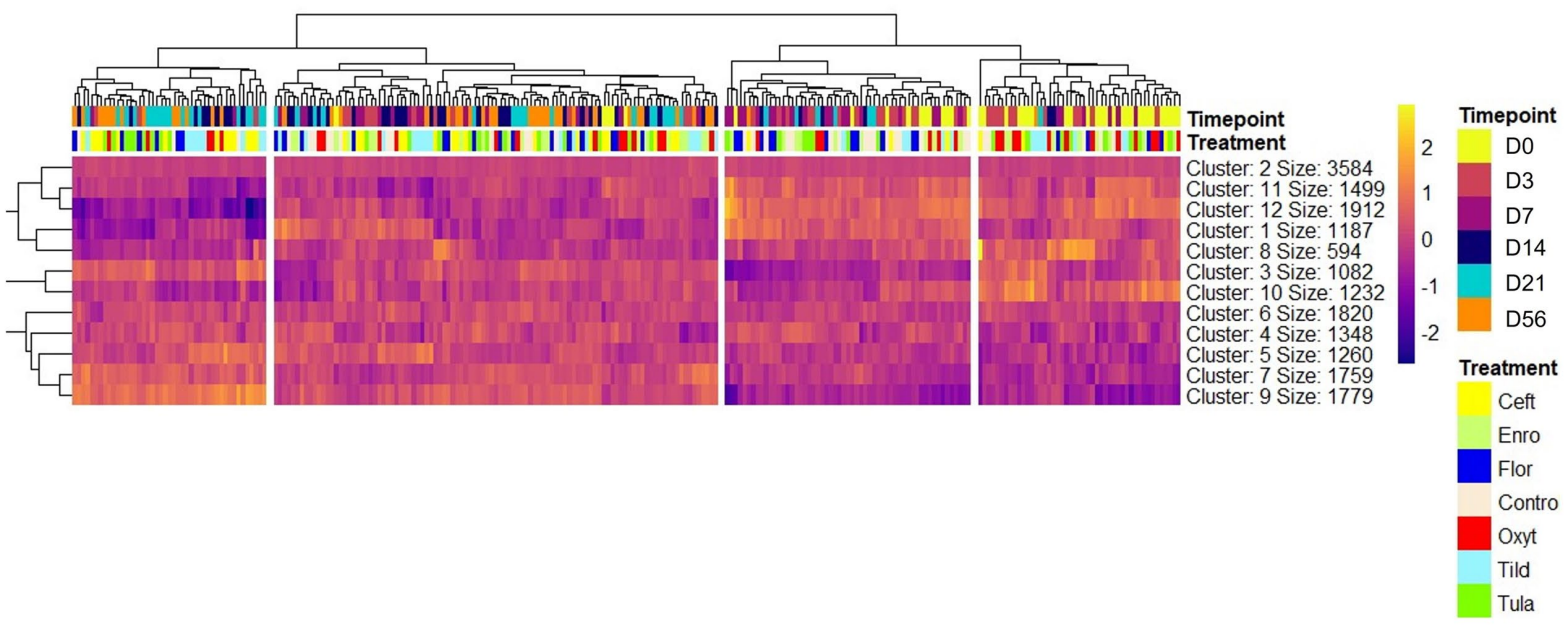
Heatmap of nine clusters of differentially expressed genes identified at all timepoints across all 252 samples. Expression patterns are shown using hierarchical clustering of genes in the rows and samples in the columns. Timepoint and treatment group are shown above the heat map under the hierarchical tree to identify the sample in each column. Gene-wise variation was standardized using z-score statistics. Color scale (yellow-to-purple) represents gene expression levels per sample; yellow and purple colors indicate increased expression and decreased expression, respectively. Note that gene hierarchical clustering of gene expression profiles segregates timepoints and treatment groups.

A total of 13,890 uniquely identified genes were differentially expressed across treatment groups and timepoints, 4,602 of which were identified as differentially expressed with both edgeR and glmmSeq (Table 1; Supplementary File S3). A heatmap was generated to visualize expression patterns across all 252 samples (Figure 1). Hierarchical clustering of samples based on expression patterns of DEGs clustered samples by timepoints with days 0, 3, and 7 as the most similar on the right and days 14, 21, and 56 grouping on the left of the map. There was no clear hierarchical clustering of treatment groups.

**Table 1:** Number of DEGs identified for each treatment group at each timepoint.

Timepoint	CEFT	ENRO	FLOR	OXYT	TILD	TULA
Day 3	411	58	28	93	1,074	0
Day 7	33	301	8	10	86	1
Day 14	29	2	7	1	116	32
Day 21	104	1	5	3	2,107	63
Day 56	1	2	3	9	13	1

### 3.2. Principal component analysis

**Figure 2:**
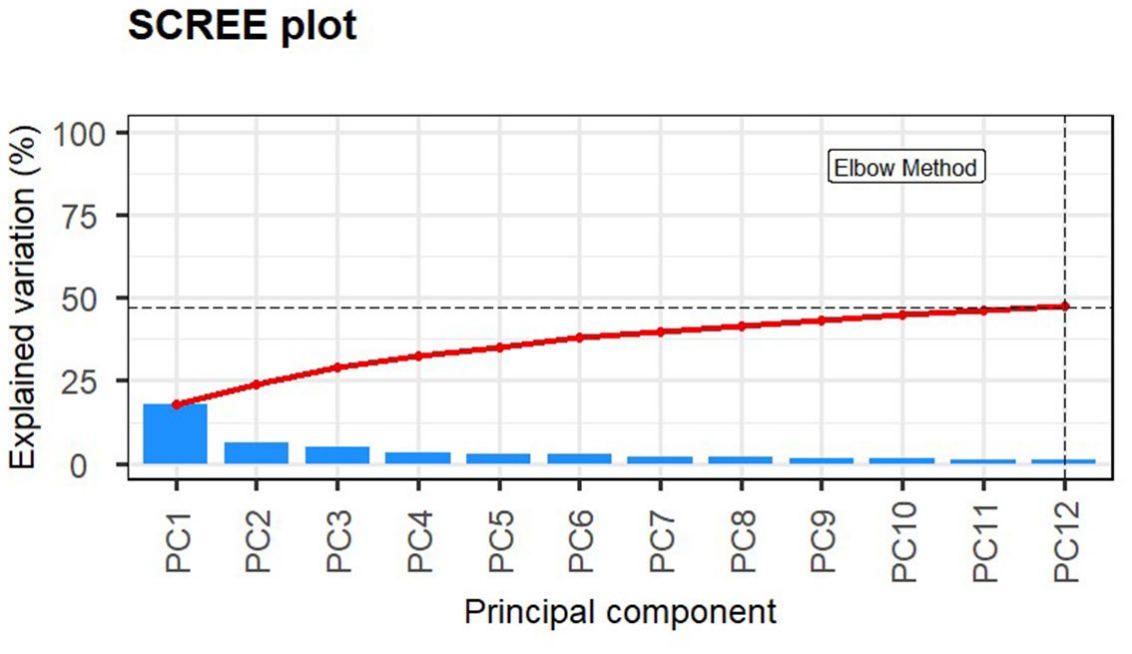
Screeplot of principle components 1:12 explaining 47.5% of the variance within the dataset. The Elbow and Horn’s parallel analysis methods were used to determine the optimum number of components to retain.

**Figure 3:**
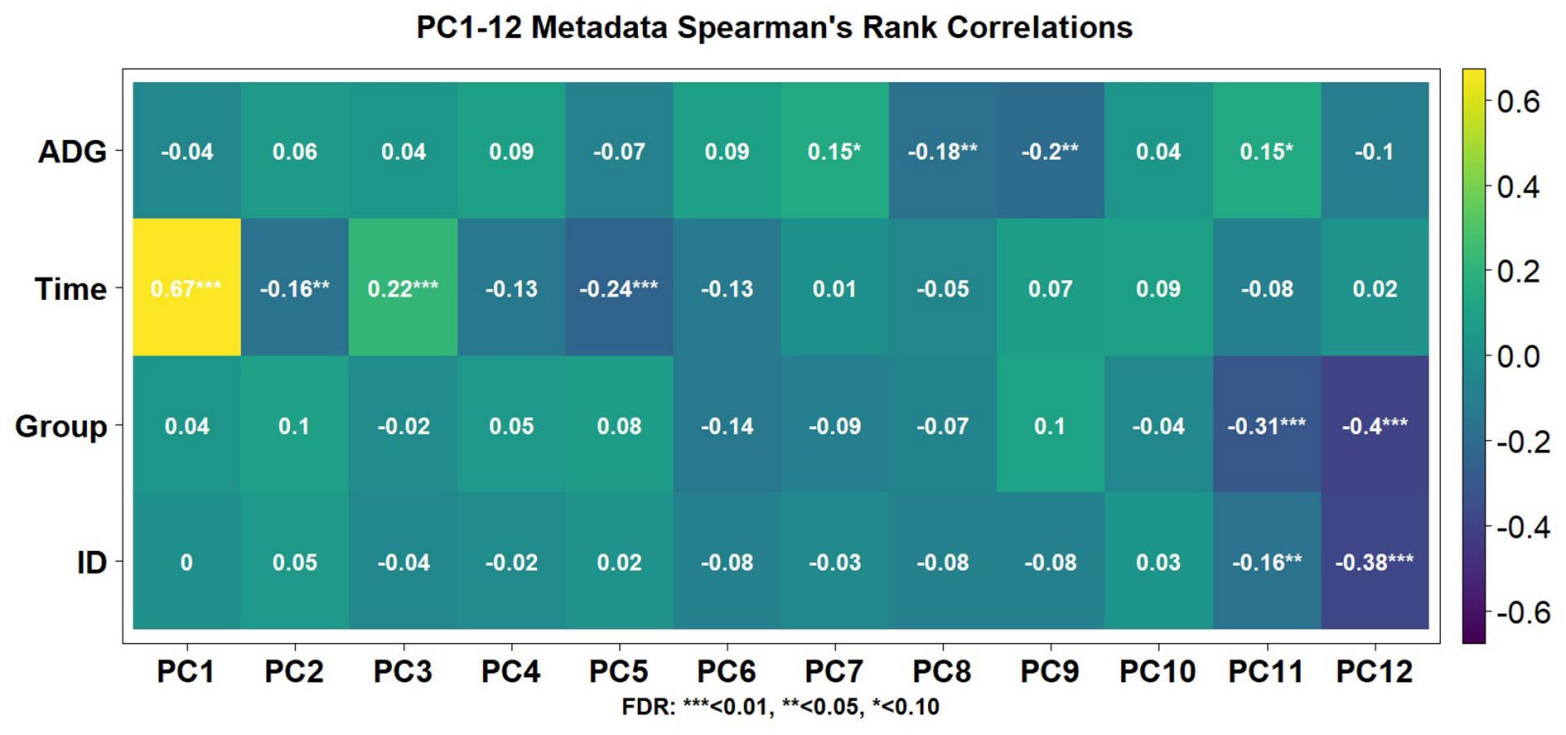
Spearman’s correlation coefficients associated with metadata components for the first 12 PCs. Each animal’s average daily gain (ADG) over the course of the trial, time, treatment group (Group), and individual animal tag number (ID) were aspects that possessed significant association with one or more PCs.

**Figure 4:**
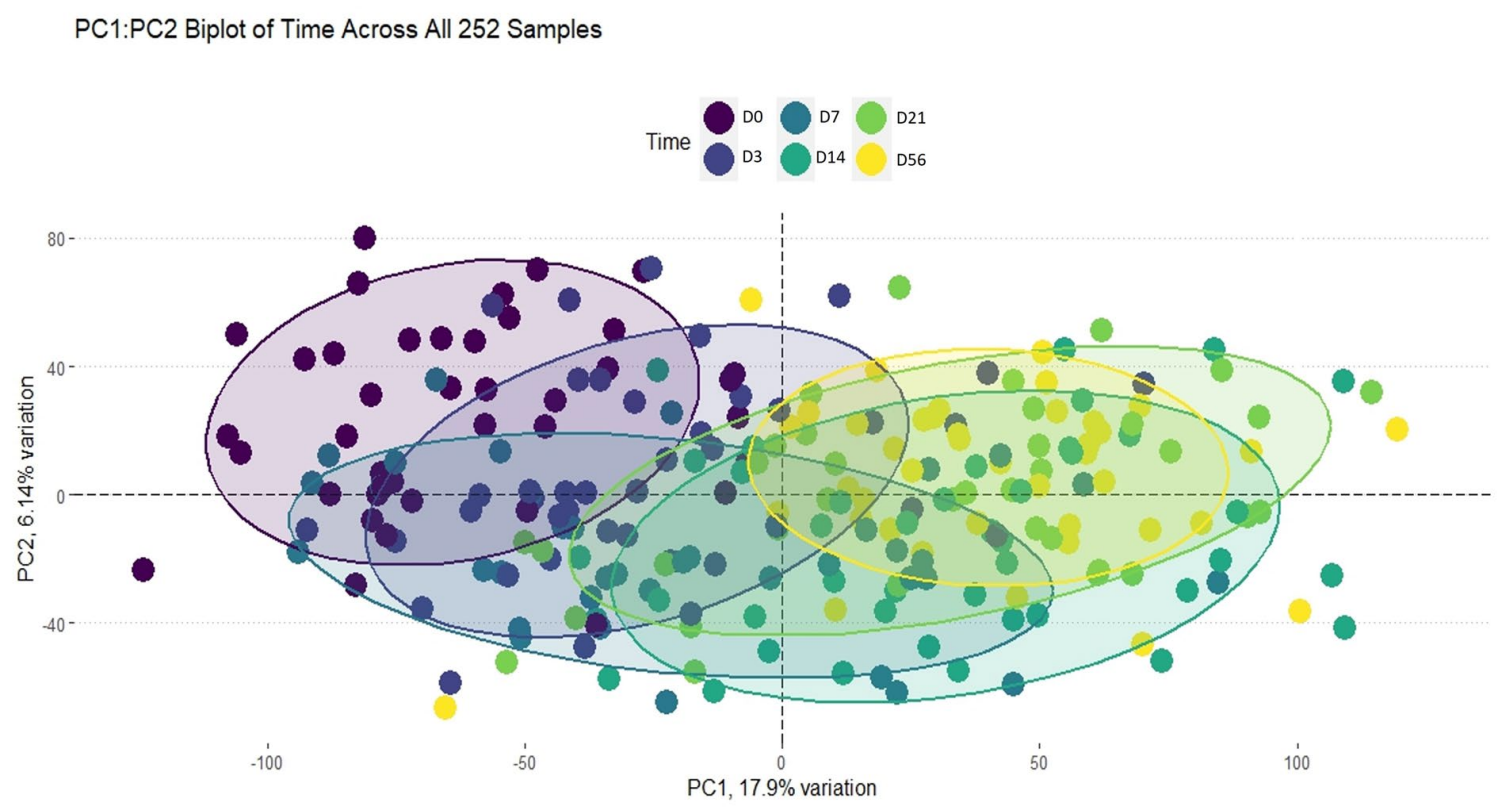
Biplot of PC1:PC2 of time across all 252 samples. The ellipses are colored by timepoint as indicated by the legend. Clustering by each timepoint can be seen, indicating time played an important role in explaining the variance within this dataset.

Using the Elbow method and Horn’s parallel analysis (34, 35), the first 12 principal components were chosen, accounting for 47% of the variance in the data as seen in Figure 2. The first principal component (PC) explained 17.9% of the variance and included the strongest and most significant correlation, which was with time (*r* = 0.67, FDR < 0.01) as shown in the Eigencorplot (Figure 3). PC2, which accounted for 6.14% of variance, was negatively correlated with time (*r* = −0.16, FDR < 0.05). Additionally, PC3 and PC5 were correlated with time as well, but in opposite directions (*r* = 0.22, *r* = −0.24, FDR < 0.01, respectively). The only PCs significantly and negatively correlated to treatment groups were PC11 (*r* = −0.31, FDR < 0.01) and PC12 (*r =* −0.4, FDR < 0.01), respectively. These two PCs were also the only components with correlations with animal ID (*r* = −0.16, *r* = −0.38, FDR < 0.05). A biplot of time across all 252 samples demonstrated the clustering of samples by timepoint as seen by the ellipses in Figure 4.

### 3.3. Pairwise intersections

**Figure 5:**
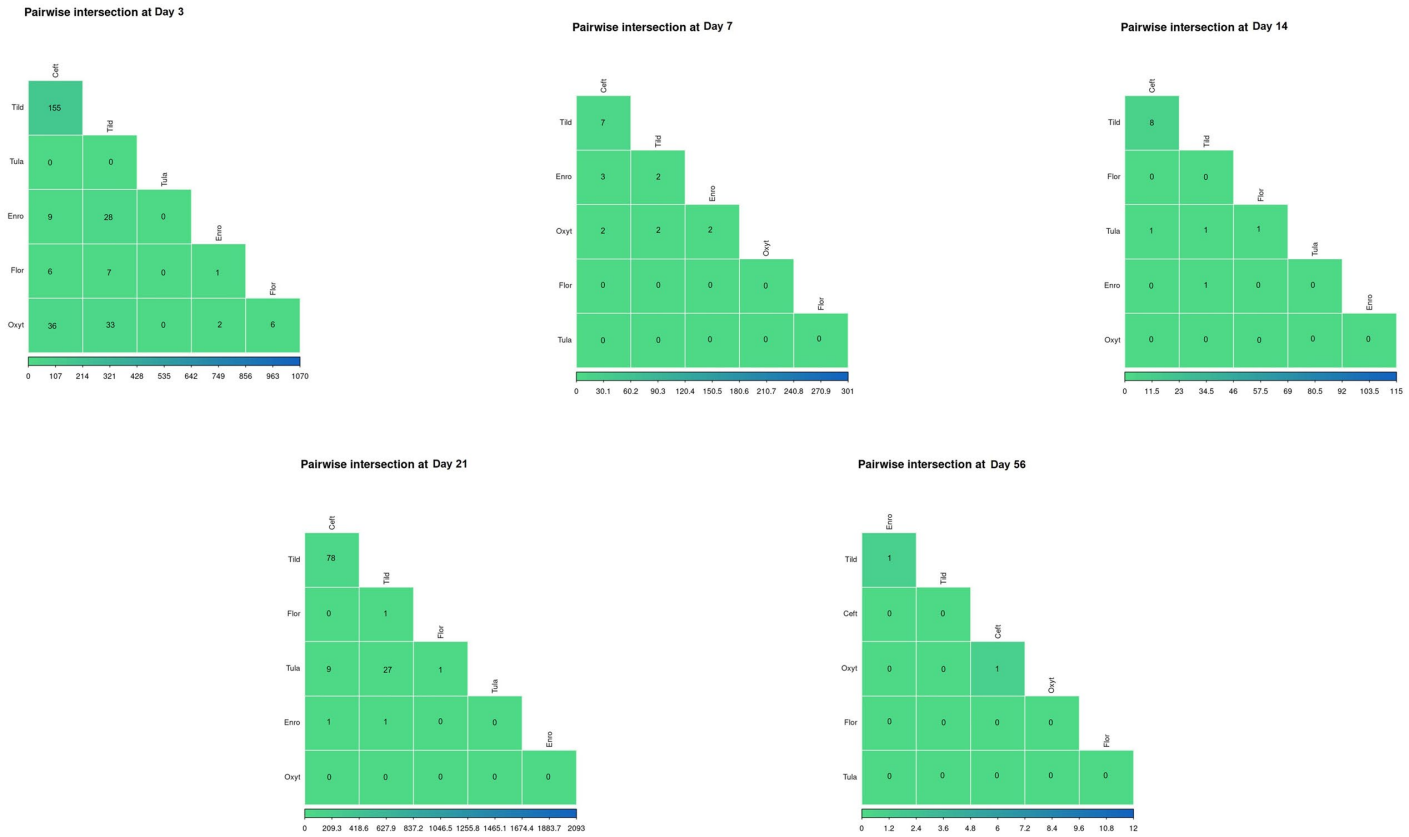
Pairwise intersections between the number of DEGs found for every treatment group at each timepoint. Color scale (green-to-blue) shows the number of DEGs in common between treatment groups; green and blue represents decreased number of overlapping DEGs and increased number of overlapping DEGs, respectively.

Pair-wise intersections of the number of DEGs shared between pairs of antimicrobial treatments at each timepoint, excluding day 0, are shown in Figure 5. There was no shared gene expression between any paired treatment combinations at day 14, day 21, and day 56. At day 3, there were minimal similarities in gene expression shared between tildipirosin and ceftiofur with between 107 and 214 DEGs shared (Supplementary File S3).

### 3.4. Gene ontology and pathway enrichment analyses

**Figure 6:**
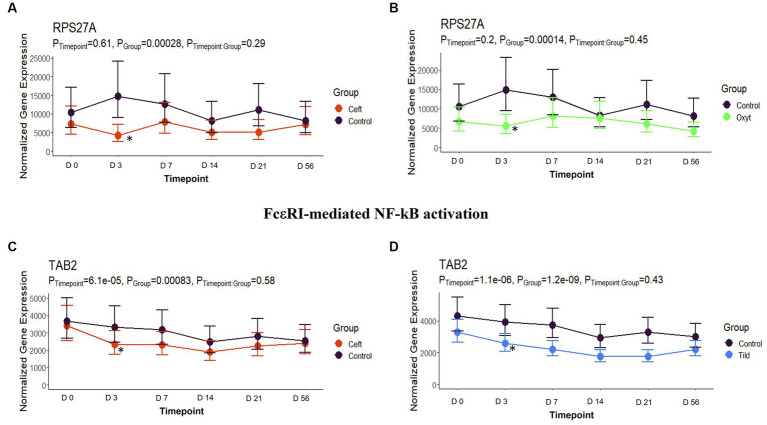
FcεRI-mediated NF- κβ activation gene expression for CEFT, OXYT, and TILD. **(A)** Trended normalized averages calculated from log_10_ transformed gene expression over time of *RSP27A* for CEFT. **(B)** Trended normalized averages calculated from log_10_ transformed gene expression over time of *RSP27A* for OXYT. **(C)** Trended normalized averages calculated from log_10_ transformed gene expression over time of *TAB2* for CEFT. **(D)** Trended normalized averages calculated from log_10_ transformed gene expression over time of *TAB2* for TILD. The dots represent the average gene expression level at that timepoint, and the bars represent the standard error of gene expression found at each timepoint in each group. NC is represented by the black lines.

**Figure 7:**
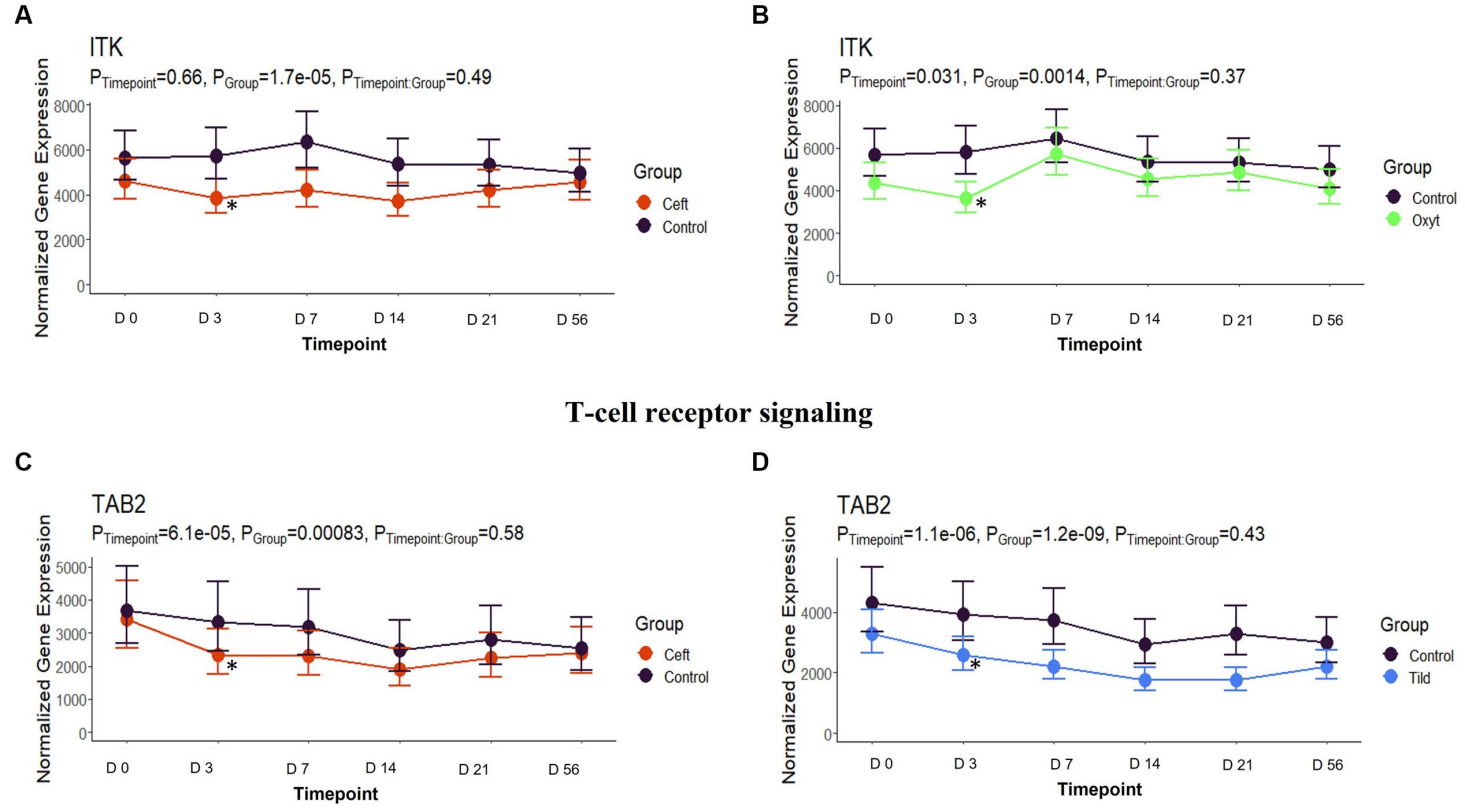
T-cell receptor signaling gene expression for CEFT, OXYT, and TILD. **(A)** Trended normalized averages calculated from log_10_ transformed gene expression over time of *ITK* for CEFT. **(B)** Trended normalized averages calculated from log_10_ transformed gene expression over time of *ITK* for OXYT. **(C)** Trended normalized averages calculated from log_10_ transformed gene expression over time of *TAB2* for CEFT. **(D)** Trended normalized averages calculated from log_10_ transformed gene expression over time of *TAB2* for TILD. The dots represent the average gene expression level at that timepoint, and the bars represent the standard error of gene expression found at each timepoint in each group. NC is represented by the black lines.

**Figure 8:**
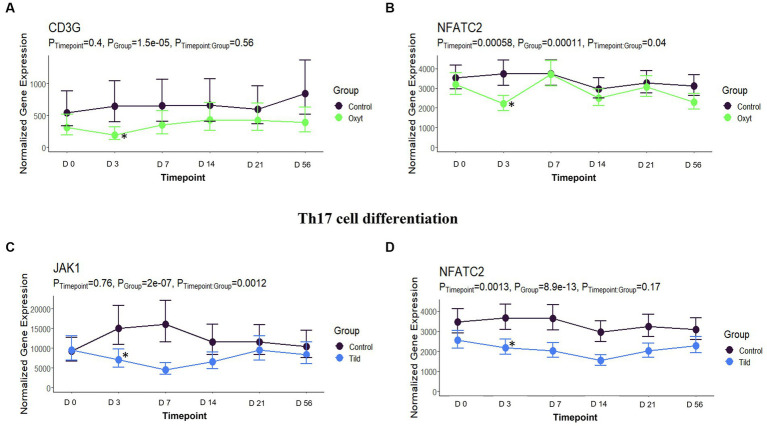
Th17 cell differentiation gene expression for OXYT and TILD. **(A)** Trended normalized averages calculated from log_10_ transformed gene expression over time of *CD3G* for OXYT. **(B)** Trended normalized averages calculated from log_10_ transformed gene expression over time of *NFATC2* for OXYT. **(C)** Trended normalized averages calculated from log_10_ transformed gene expression over time of *JAK1* for TILD. **(D)** Trended normalized averages calculated from log_10_ transformed gene expression over time of *NFATC2* for TILD. The dots represent the average gene expression level at that timepoint, and the bars represent the standard error of gene expression found at each timepoint in each group. NC is represented by the black lines.

When compared only to NC, unique DEGs were identified for CEFT (*n* = 526), ENRO (*n* = 340), FLOR (*n* = 56), OXYT (*n* = 111), TILD (*n* = 3,001), and TULA (*n* = 87). At day 0, there were no identifiable functional enrichment terms (Tables 2, 3). At day 3, in comparison to NC, there were 44 pathways and 49 GO terms that were enriched for CEFT DEGs (*n* = 411). For ENRO, 58 DEGs enriched for two pathways and six GO terms at day 3. There were 28 unique DEGs for FLOR that did not enrich any pathways or GO terms at day 3. For OXYT, 93 DEGs were identified with 67 pathways and 33 GO terms enriched. With the greatest number of DEGs identified overall, there were 1,074 unique DEGs identified for TILD with a total of 146 pathways and 170 GO terms enriched at day 3. There were no DEGs found for TULA shared between edgeR and glmmSeq analyses at day 3. CEFT, TILD, and OXYT shared multiple functional enrichment pathways related to FcεRI-mediated NF-kB activation and T-cell receptor signaling, which were downregulated in these three treatment groups compared to NC at day 3 as seen in Figures 6, 7, respectively. For the latter pathway, CEFT shared genes with OXYT and TILD that were downregulated which included *RPS27A* and *TAB2*, respectively. Genes uniquely identified for CEFT for T-cell receptor signaling included *TAB2, RPS27A, ITK, MALT1*, and *SKP1*. For this pathway, OXYT had two genes matching CEFT’s of the five that were downregulated: *ITK, RPS27A, PRKCQ, ZAP70*, and *CD3G*. Genes downregulated in the TILD cattle included *TAB2, NFKBIA, CUL1, CHUK, PAG1, BCL10*, and *NCK1*. OXYT and TILD shared one gene, *NFATC2*, related to Th17 cell differentiation at day 3 as seen in Figure 8. Complete findings of functional enrichment analyses at day 3 are found in Supplementary File S4.

**Table 2:** Number of enriched pathways identified for each treatment group at each timepoint.

Timepoint	CEFT	ENRO	FLOR	OXYT	TILD	TULA
Day 3	44	2	0	67	146	0
Day 7	31	42	6	6	9	0
Day 14	7	0	7	0	4	232
Day 21	1	0	132	0	162	8
Day 56	0	0	127	23	9	0

**Table 3:** Number of gene ontology terms identified for each treatment group at each timepoint.

Timepoint	CEFT	ENRO	FLOR	OXYT	TILD	TULA
Day 3	49	6	0	33	170	0
Day 7	7	116	100	24	1	0
Day 14	15	0	83	0	5	86
Day 21	1	0	53	0	185	4
Day 56	0	0	53	35	36	0

**Figure 9:**
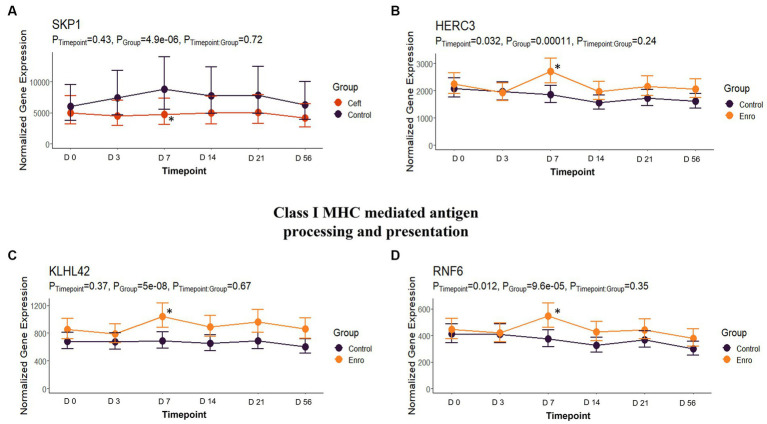
Class I MHC mediated antigen processing and presentation gene expression for CEFT and ENRO. **(A)** Trended normalized averages calculated from log_10_ transformed gene expression over time of *SKP1* for CEFT. **(B)** Trended normalized averages calculated from log_10_ transformed gene expression over time of *HERC3* for ENRO. **(C)** Trended normalized averages calculated from log_10_ transformed gene expression over time of *KLHL42* for ENRO. **(D)** Trended normalized averages calculated from log_10_ transformed gene expression over time of *RNF6* for ENRO. The dots represent the average gene expression level at that timepoint, and the bars represent the standard error of gene expression found at each timepoint in each group. NC is represented by the black lines.

**Figure 10:**
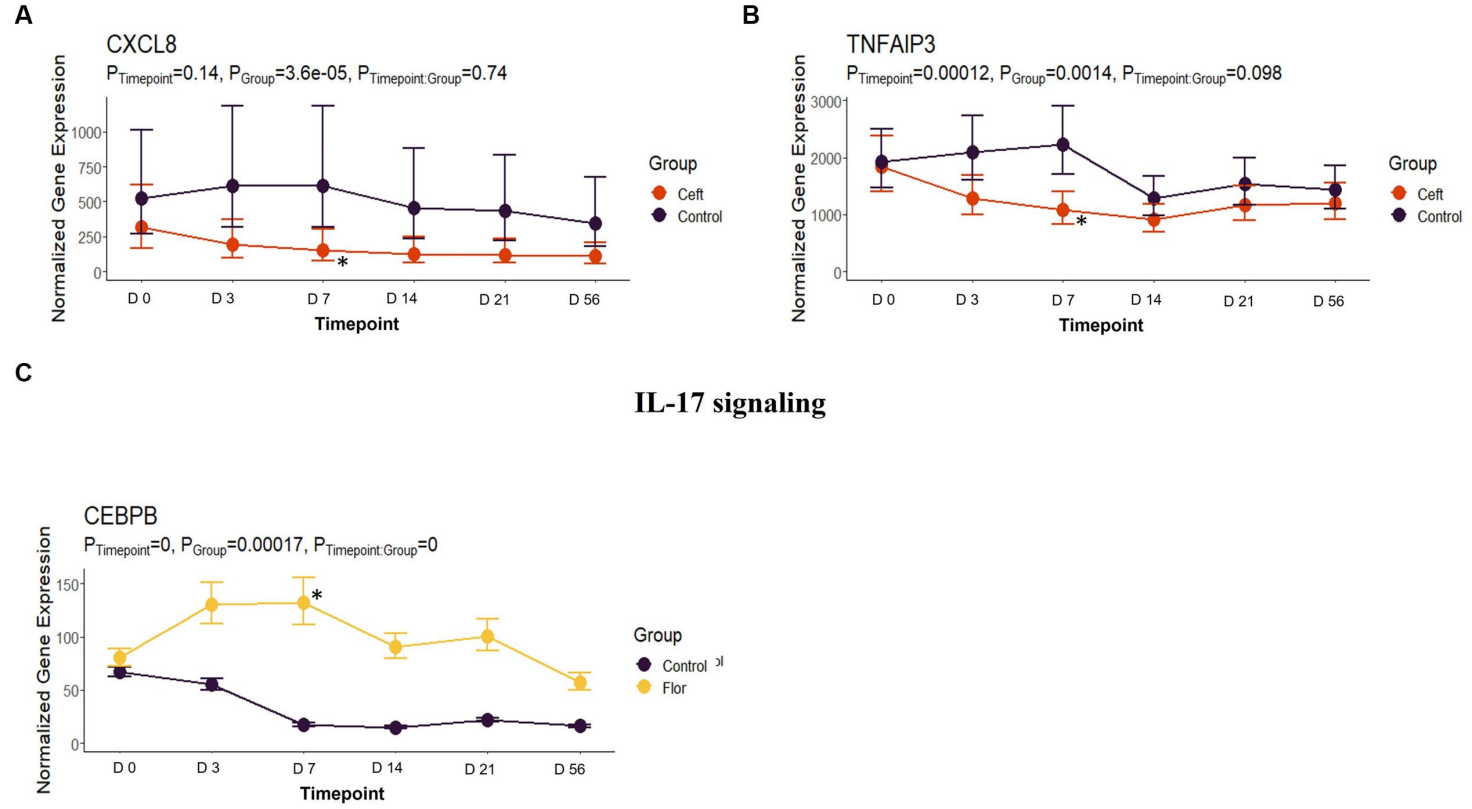
IL-17 signaling gene expression for CEFT and FLOR. **(A)** Trended normalized averages calculated from log_10_ transformed gene expression over time of *CXCL8* for CEFT. **(B)** Trended normalized averages calculated from log_10_ transformed gene expression over time of *TNFAIP3* for CEFT. **(C)** Trended normalized averages calculated from log_10_ transformed gene expression over time of *CEBPB* for FLOR. The dots represent the average gene expression level at that timepoint, and the bars represent the standard error of gene expression found at each timepoint in each group. NC is represented by the black lines.

In comparison to NC cattle at day 7, CEFT had 33 DEGs that enriched 31 pathways and seven GO terms. With the greatest number of DEGs identified at this timepoint, ENRO had 301 unique DEGs with 42 pathways and 116 GO terms enhanced. There was a decrease in DEGs in FLOR with eight uniquely identified genes that enriched six pathways and 100 GO terms. Similarly, OXYT had 10 DEGs that enriched six pathways and only 24 terms at day 7. For TILD, 86 DEGs were found that enriched nine pathways and one GO term, which was the positive regulation of T-cell apoptotic process. There were not enough DEGs (*n* = 1) shared between edgeR and glmmSeq results to perform functional enrichment on TULA at day 7. At this timepoint, ENRO and CEFT enriched for Class I major histocompatibility complex (MHC) mediated antigen processing and presentation pathways (Figure 9), while CEFT and FLOR had DEGs that affected IL-17 signaling pathways (Figure 10). For the pathway related to Class I MHC, DEGs identified for CEFT, *FBXO9* and *SKP1*, were downregulated while DEGs for ENRO (*TRIP12, HECTD1, ASB7, KLHL42, HERC3, RNF6*, and *CUL1*) were upregulated. Similarly, DEGs for CEFT were downregulated compared to upregulated DEGs for FLOR related to an IL-17 signaling pathway. CEFT genes included *CXCL8* and *TNFAIP3*, and one DEG was identified for FLOR in relation to this pathway, *CEBPB*. Complete findings of functional enrichment analyses at day 7 are found in Supplementary File S5.

**Figure 11:**
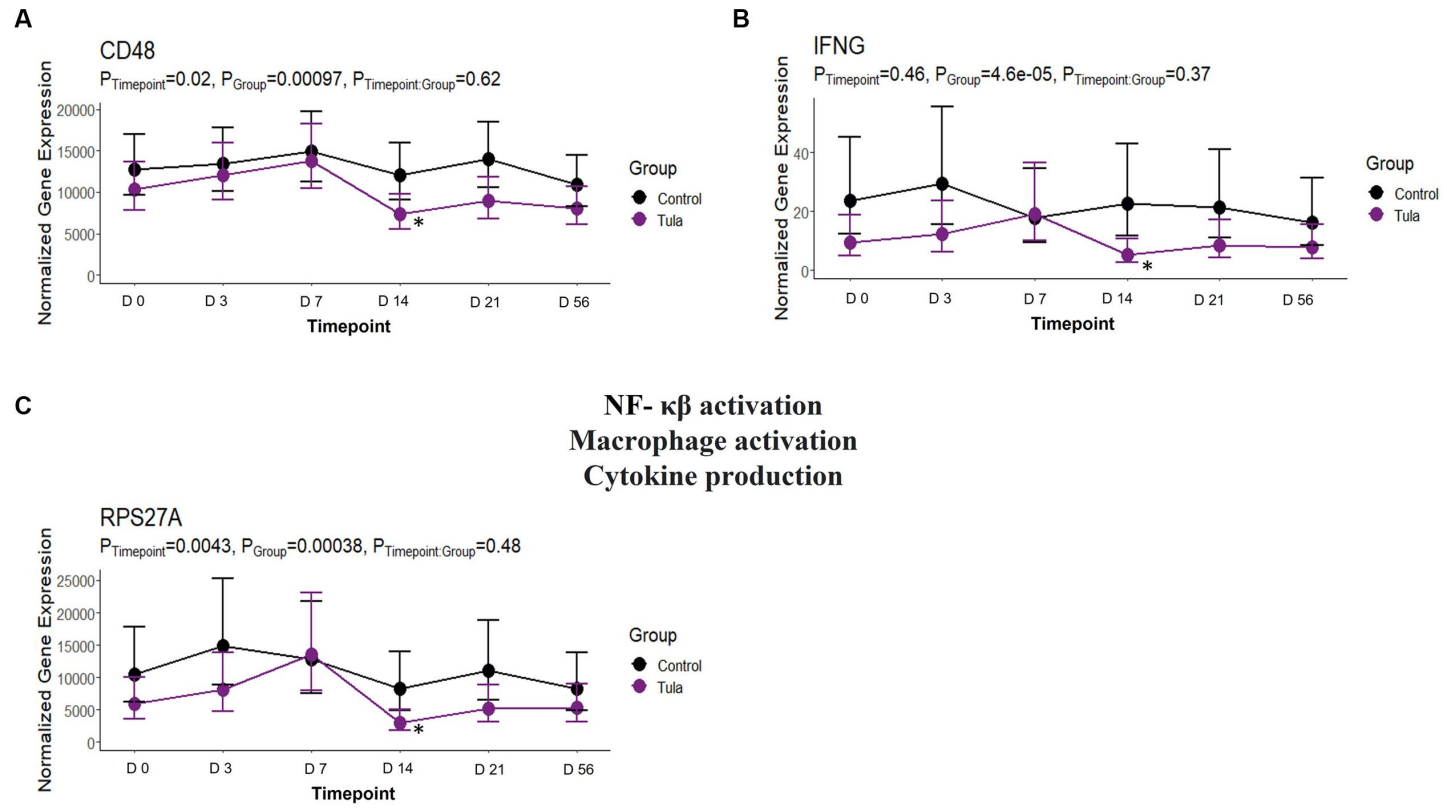
Gene expression downregulated by TULA at T4, day 14, related to NF – κβ activation, macrophage activation, and cytokine production. **(A)** Trended normalized averages calculated from log_10_ transformed gene expression over time of *CD48* for TULA. **(B)** T Trended normalized averages calculated from log_10_ transformed gene expression over time of *IFNG* for TULA. **(C)** Trended normalized averages calculated from log_10_ transformed gene expression over time of *RPS27A* for TULA. The dots represent the average gene expression level at that timepoint, and the bars represent the standard error of gene expression found at each timepoint in each group. NC is represented by the black lines.

At day 14, there were 29 unique DEGs identified for CEFT with seven and 15 pathways and GO terms enriched, respectively. There were not enough DEGs (*n* = 2) shared between edgeR and glmmSeq analyses for ENRO at day 14 to perform functional enrichment. For FLOR, seven DEGs that enriched for seven pathways and 83 GO terms were identified. There were not enough DEGs (n = 1) identified at day 14 for OXYT to perform functional enrichment. TILD had 116 unique DEGs that enriched four pathways and five GO terms at this timepoint. For TULA, 32 DEGs were identified, and the most pathways at 232 and 86 terms were enriched. No shared pathways between treatments were identified. There were 19 pathways and eight GO terms enriched for TULA related to NF- κβ activation, and interleukin and interferon signaling. Three genes were enriched for these pathways and included *RPS27A, IFNG*, and *CD48*. See Figure 11. Complete findings of functional enrichment analyses at day 14 are found in Supplementary File S6.

**Figure 12:**
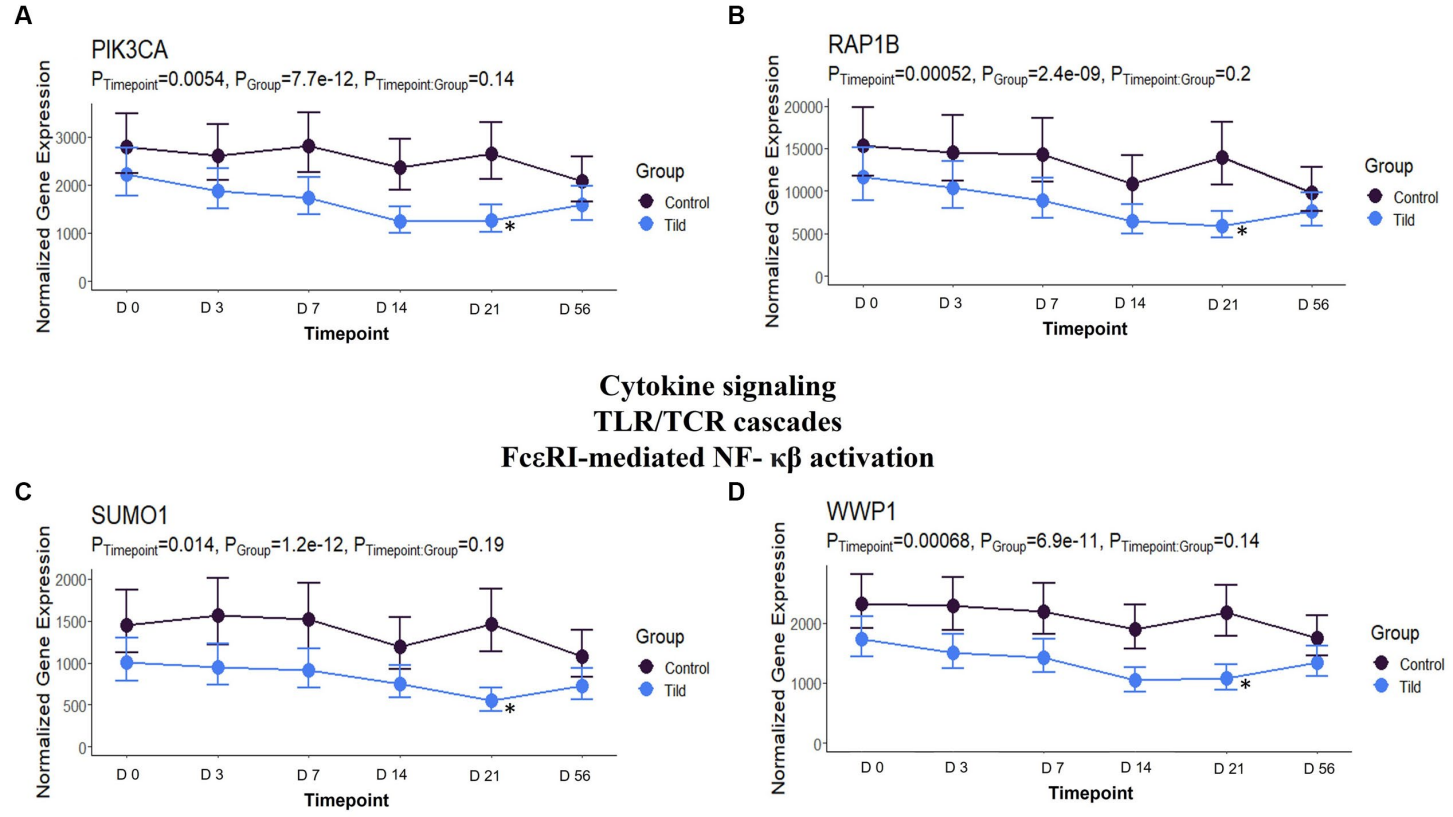
Gene expression downregulated by TILD at T5, day 21, related to cytokine signaling, Toll-like receptor/T-cell receptor cascades, and FcεRI-mediated NF- κβ activation. **(A)** Trended normalized averages calculated from log_10_ transformed gene expression over time of *PIK3CA* for TILD. **(B)** Trended normalized averages calculated from log_10_ transformed gene expression over time of *RAP1B* for TILD. **(C)** Trended normalized averages calculated from log_10_ transformed gene expression over time of *SUMO1* for TILD. **(D)** Trended normalized averages calculated from log_10_ transformed gene expression over time of *WWP1* for TILD. The dots represent the average gene expression level at that timepoint, and the bars represent the standard error of gene expression found at each timepoint in each group. NC is represented by the black lines.

When compared to NC, 104 unique DEGs were identified for CEFT at day 21, and they enriched for one Reactome pathway and one GO term. There were not enough DEGs (*n* = 1) shared between edgeR and glmmSeq for ENRO at day 21 to perform functional enrichment. FLOR had five DEGs that enriched for 132 pathways and 53 GO terms at this timepoint. OXYT had three DEGs identified, but they could not be annotated to perform functional enrichment. Once again, with the largest number of DEGs, 2,107 genes were identified for TILD and enriched for 162 pathways and 185 GO terms. TULA had 63 unique DEGs in common between edgeR and glmmSeq that enriched eight pathways and four GO terms. There were no shared pathways between any treatment groups. At day 21, TILD had 13 enriched pathways related to cytokine signaling in the immune system as shown in Figure 12. Complete findings of functional enrichment analyses at day 21 are found in Supplementary File S7.

At day 56, CEFT and ENRO did not have enough DEGs (n = 1, n = 2, respectively) to perform functional enrichment. However, FLOR had three DEGs that enriched for 127 pathways and 53 GO terms. There were nine DEGs for OXYT that enriched 23 pathways and 35 GO terms. For TILD, there were 13 DEGs shared between analyses that coded for nine pathways and 36 GO terms. TULA only had one DEG identified that did not enrich any pathways or terms. There were no specific pathways shared among treatment groups at this timepoint. Complete findings of functional enrichment analyses at day 56 are found in Supplementary File S8.

For timepoint:group interactions, unique DEGs (FDR ≤ 0.05) were found for CEFT (*n* = 339), ENRO (*n* = 257), FLOR (*n* = 319), OXYT (*n* = 236), TILD (*n* = 373), and TULA (*n* = 276) (Supplementary File S3). Functional enrichment was performed for each timepoint:group comparison. There were 14 pathways and 32 GO terms for CEFT, primarily related to cell scavenging, cell surface activity and reception, ion binding and transport, and sarcomere and myosin activity. For ENRO, there were 6 pathways and 13 GO terms enriched, including the upregulation synthesis of prostaglandins and thromboxanes, chloride and calcium channel/cell membrane activity, and cell membrane synapse and depolarization. For FLOR, there were 2 pathways and 15 GO terms, related to lipopolysaccharide binding, hydrogen peroxide biosynthesis, collagen and intermediate filament organization, and defense against gram-negative bacteria. For OXYT, there was enrichment for only 1 pathway and 3 GO terms, of which were relatively non-specific and unrelated to immunomodulation. For TILD, there were 4 pathways and 14 GO terms enriched, related to the positive regulation of neutrophil chemotaxis, extracellular matrix structure and receptor interaction, and autophagy. For TULA, no pathways were enriched, and there were 11 GO terms, which corresponded with carbohydrate binding, ion transport and binding, extracellular matrix structure, and defense response against gram-positive bacterium. Complete findings of functional enrichment analyses for timepoint:group interactions for all treatments across all timepoints are found in Supplementary File S9.

## 4. Discussion

Previous research has sufficiently employed the use of transcriptomics and RNA-Seq in the exploration of predictive biomarkers for BRD, the pathogens involved in development of this disease, and the presence of antimicrobial resistance genes (ARGs) of the bacterial pathogens associated with BRD. Trials utilizing tissue samples such as lung, lymph nodes, and tonsils have demonstrated how each viral and bacterial agent acts as a pathogen, causing clinical disease (11, 12). More recently, the whole blood transcriptome has been investigated for potential predictive molecules, protective immunological mechanisms, and regulatory patterns of BRD in beef cattle (13, 14, 36, 37). Whole genome sequencing has also been used to examine bacterial transcriptome profiles and identify ARGs and the drug classifications of which species have developed resistance (10, 38, 39). Although the findings mentioned above are pertinent to attempt to understand BRD and involved pathogens, there is a lack of transcriptome research examining the relationship between antimicrobials and their effects on the host immunological response. Identifying mechanisms and potential secondary properties of antimicrobials is important to understanding how drugs maintain efficacy despite the rise of AMR.

In this study, we identified 34,267 DEGs associated by day (i.e., time) and 1,800 DEGs associated with the interaction of group and day in glmmSeq (Supplementary File S3). Based on hierarchical clustering of total gene expression across all samples (Figure 1), time was a major component in driving discernible gene expression patterns; days 0, 3 and 7 clustered while days 14, 21, and 56 shared more similar gene expression patterns. The biplot of PC1:PC2 also demonstrated the importance of time within this dataset as seen by the clustering of the ellipses by timepoint (Figure 4). Time is shown to biologically factor in the development of the immune system, where progression of the immune systems in calves occurs in small steps from conception to maturity at approximately 6 months after birth (40). The cells associated with innate immunity, such as neutrophils, macrophages, and natural killer cells, are influenced by age and time. While neutrophil counts may be higher in neonatal calves, their phagocytic abilities are reduced compared to older calves, as are macrophages (41). Neutrophil activity increases to adult levels by 5 months of age (40). When calves enter feedlots at lower weights and younger ages, they are predisposed to BRD, largely due to the fact their immune system is not always fully developed. In cattle, most of the immune system maturity is seen between five and 8 months of age. While this does not mean that young calves are not capable of responding to antigens, the response may be weaker and thus, easier for pathogens to overcome (42). With the addition of novel pathogens, their immune systems fail to produce a sufficient response to protect them from developing BRD upon arrival to a stocker or feedlot facility (42). Previous research suggests that peak clinical signs of disease occur 7 to 14 days after peak exposure to viral and bacterial pathogens (3, 43, 44). There are other factors that potentially explain the effect time had on this dataset, such as physiological stress and acclimation. The effect physiological stress can have on a calf’s immune system can lead to prolonged activation of the hypothalamus-pituitary–adrenal (HPA) axis, suppressing the immune system and increasing susceptibility to disease (45). Additionally, acclimation to a new environment and exposure to novel pathogens is a hypothesis to consider. In response to this acclimation, many calves’ immune systems are unable to mount an appropriate response. Therefore, time plays an important role in host immunological response to antimicrobials administered metaphylactically.

In the present study, we identified 4,602 DEGs across the six antimicrobials administered (Supplementary File S3; Table 1). Regarding functional enrichment of these DEGs (Supplementary Files S4–S8), the major functions found across treatments included cytokine signaling and T-cell receptor signaling pathways. There was no hierarchical clustering by treatment group (Figure 1) and no correlations between groups as shown by the pairwise intersection plots (Figure 5), suggesting each antimicrobial acts through various mechanisms controlled by different DEGs. At day 3, FcεRI-mediated NF- κβ activation pathways were enriched for CEFT, OXYT, and TILD. As supported by previous literature, ceftiofur and oxytetracycline inhibited NF- κβ activation by reducing the translocation of NF- κβ from the cytoplasm to the nucleus and *via* phosphorylation, respectively (46, 47). CEFT and OXYT had one DEG in common to regulate this pathway even though they affect NF- κβ through different mechanisms: *RPS27A*. To our knowledge, there is no published literature regarding tildipirosin’s downregulation of FcεRI-mediated NF- κβ activation. However, clarithromycin has been reported to inhibit NF- κβ activation in human peripheral blood mononuclear cells and pulmonary epithelial cells (48). TILD and CEFT share a DEG (*TAB2*) that enriches this pathway (Figure 6). It is inappropriate to assume that they use the same mechanism though due to the lack of published literature regarding tildipirosin’s effect on FcεRI-mediated NF- κβ activation.

Downregulation of T-cell receptor signaling also occurred at day 3 by CEFT, OXYT, and TILD. The influence of ceftiofur on T-cells and B-cells has been investigated in chicks, and it was found that the percentages of both types of cells were decreased (49). A change in the specific T-cell type ratio was also observed with decreased percentages of CD4^+^ CD8^−^ cells (49). A study conducted by Platania et al. (50) demonstrated that oxytetracycline reduced the proliferative response of T lymphocytes through an *in vivo* model of autoimmune disease related to glucocorticoid-induced tumor necrosis factor receptor-related gene (GITR; TNFSRF18) in mice. While tildipirosin was not specifically mentioned, another macrolide (rapamycin) was reported to interfere with signal transduction pathways required for T-cell activation and growth in various species such as mice, pigs, and monkeys (50). The inhibitory effects of rapamycin on T-cell activation were mediated through the formation of pharmacologically active complexes with members of a family of intracellular receptors that are signaling proteins required for T-cell antigen receptor engagement (50).

The final pathway enriched among multiple treatments at day 3 is related to T-helper (Th) 17 cell differentiation. OXYT and TILD have one DEG in common for this pathway out of four and seven genes, respectively (NFATC2). While, to our knowledge, there is no published literature regarding oxytetracycline and Th17 cell differentiation, there has been a report of another tetracycline: minocycline. Minocycline treatment in mice reduced the production of interleukin (IL)-17, and a positive correlation between glucocorticoid-induced tumor necrosis factor receptor-related gene (GITRL) signaling and Th17 induction has been reported in previous literature (51, 52). Tang and colleagues (52) demonstrated that IL-17 production and Th17 differentiation was triggered by *GITLR* intracellular signaling. Similarly, there was no previous research for tildipirosin’s effect on Th17 differentiation. However, clarithromycin, another macrolide, was investigated when treating mice infected with macrolide antibiotic-resistant *Streptococcus pneumoniae* (53). Clarithromycin administration impaired the frequency and number of Th17 cells within the lungs of infected mice. Although the cattle treated in the current trial were healthy, metaphylaxis is applied to cattle at high risk of developing BRD. Therefore, previous literature investigating antimicrobials and their interactions with bacterial or viral pathogens is highly relevant to understand the importance of mechanisms found by this study.

Class I MHC mediated antigen processing and presentation was a pathway enriched by CEFT and ENRO on day 7, but the drugs regulated the DEGs in opposite directions. Ceftiofur downregulated two genes while enrofloxacin upregulated seven. Another third-generation cephalosporin, ceftriaxone, has been reported to affect antigen presentation by binding directly to the immunogenic peptide embedded in MHC or to cell surface proteins, preventing necessary processing before the presentation (54, 55). This could potentially be the mechanism behind the downregulation of genes in the CEFT cattle. The genes upregulated by enrofloxacin were all related to ubiquitination and proteasome degradation, which play a central role in generating class I MHC antigens. Intracellular foreign or deviated host proteins are cleaved into peptide fragments so they can be loaded on to class I MHC molecules and presented to cytotoxic T-cells (56).

Similarly, the DEGs enriching the pathway for IL-17 signaling were downregulated in CEFT and upregulated in FLOR cattle. No DEGs were shared between the two independent comparisons, suggesting different genomic processes were in use to regulate IL-17. Interleukin-17 is a key cytokine that links T-cell activation to neutrophil mobilization and activation, and IL-17 mediates innate immunity to pathogens or contribute to the pathogenesis of inflammatory disease (57). In previous literature, cefazolin, a first-generation cephalosporin antibiotic, was shown to interact with IL-15-dependent TNF-α and IL-17 synthesis (58). Cefazolin reduced production of IL-17, among other various cytokines (58). No previous research was identified corroborating the findings of florfenicol’s upregulation of *CEBPB* in relation to IL-17, possibly illustrating a novel secondary mechanism for this antimicrobial. The same gene, *CEBPB*, also influenced a tumor necrosis factor (TNF) signaling pathway and a GO term of “positive regulation of inflammatory response.”

There were no shared pathways identified at day 14 between any antimicrobials, but TULA possessed 19 pathways and 8 GO terms enriched related to NF- κβ activation, macrophage activation and differentiation, and production of various cytokines such as IL-3, IL-5, and IFN-γ. There were three genes acting as the main drivers of the pathways and terms: *RPS27A*, *IFNG*, and *CD48*. It has been reported that tulathromycin significantly inhibits proinflammatory NF- κβ signaling in bovine neutrophils and reduced secretion of proinflammatory CXCL-8 in lipopolysaccharide (LPS)-stimulated macrophages (59, 60). In another trial, the effect of tulathromycin was examined in a nonbacterial *in vivo* model of pulmonary inflammation, where tulathromycin administration inhibited phospholipases and altered leukotriene B_4_, prostaglandin E_2_, and lipoxin A_4_ production (61). The findings from the present trial support the anti-inflammatory benefits of tulathromycin while providing insight on the DEGs being downregulated to inhibit pathways discussed above. Furthermore, tulathromycin has been shown to have immunomodulatory effects in species other than cattle, such as rabbits, pigs, and horses (62–64).

On day 21, there were no shared pathways or GO terms between treatment groups. TILD possessed the most DEGs identified, and pathways/GO terms enriched at 2,107 and 347, respectively. Pathways found were related to cytokine signaling, toll like receptor (TLR)/TCR cascades, and FcεRI-mediated NF-κβ activation. Genes downregulating these pathways included *FBX09*, *PIK3CA*, *RAP1B*, *RNF14*, *SUMO1*, *UBE2Q2*, and *WWP1*. Although minimal published literature exists regarding the effect of tildipirosin specifically on cytokines and TCR/TLR cascades, there is evidence that macrolides can interfere with signal transduction of T-cell activation and growth, and the impairment of T-cell production thus decreasing the immune response, shown through trials completed with rapamycin and clarithromycin (50, 53).

On day 56, there were 25 DEGs in total identified across three treatment groups (FLOR, OXYT, and TILD) when compared to NC. However, none of the genes that enriched pathways related to immune function were differentially expressed. This may demonstrate a transient induction or influence of genomic regulation by these drugs, as the majority of the antimicrobials used in this trial possess half-lives less than 3 days, with the exception of tildipirosin which is 9 days in the plasma and 10 days in the lung (65).

To investigate the intersection of time and treatment within this study, we identified several DEGs and functional enrichments within each group comparison. Interestingly, we identified direct immune-related mechanisms in five of the six groups: CEFT, ENRO, FLOR, TILD, and TULA (Supplementary File S9). The GO terms and pathways identified for CEFT primarily related to ion reception and cell scavenging. One study, albeit in combination with vanadium and performed *in vitro*, suggested that cephalosporin interacts with tissue macrophages and act as a reducing scavenger (66). Of particular interest, DEGs identified within ENRO enriched for prostaglandin and thromboxane syntheses and arachidonic acid metabolism. In recent studies, it has been shown that danofloxacin administration, a closely related fluoroquinolone drug, significantly reduced prostaglandin E2 levels and induced lipid peroxidation (67, 68). From the FLOR enrichment analysis, we identified significant terms and pathways related to lipopolysaccharide (LPS) binding and gram-negative bacterium defense, primarily driven by *LBP*, several members of the cathelicidin family of antimicrobial peptides (*CAMP*, *CATHL1*, and *CATHL2*), and the adhesion G protein-coupled receptor gene *ADGRB1*. Several studies have suggested that florfenicol helps combat bacterial infection, regulate LPS-induced immune proliferation, and quell host inflammation *via* these mechanisms in a concentration-dependent manner (69–72). For both TILD and TULA interaction analyses, we identified enrichment for neutrophil chemotaxis and autophagy, extracellular matrix structure, and defense against bacterium. A recent therapeutic review article related to adjunct therapies for SARS-CoV-2 infection highlights that clinical use of azithromycin, a closely related macrolide, appears to regulate proinflammatory cytokine production, inhibit neutrophilic infiltration, and alters autophagosomes within macrophages (73). While unclear at this time, this may suggest that these macrolides alter neutrophilic release of proinflammatory cytokines, such as IL-1β, and enhance leukocyte stimulation through autophagy (74, 75).

One limitation of this study was the variation of breeds within the sample population; however, randomization was used to successfully account for this and other potential confounders. Other important confounders such as BW and feed were controlled by the experimental design. This aspect could account for variation biologically between samples. Additionally, there was only one pen per treatment. The lack of replication reduces the ability to extrapolate results to other populations, but these findings provide a direction for further research to explore the mechanisms found for each antimicrobial. A strength of this trial was the detection of DEGs using two different statistical methods, showing the robustness of the results.

## Data availability statement

The data presented in the study are deposited in the National Center for Biotechnology Information Gene Expression Omnibus (NCBI-GEO), accession number GSE225025. R code for statistical analyses of raw gene count data is found at https://github.com/mscott16/metamultiome.

## Ethics statement

The animal study was approved by West Texas A&M University (WTAMU) Institutional Animal Care and Use Committee. The study was conducted in accordance with the local legislation and institutional requirements.

## Author contributions

RB: Data curation, Formal analysis, Investigation, Methodology, Validation, Visualization, Writing – original draft, Writing – review & editing. JR: Conceptualization, Funding acquisition, Investigation, Methodology, Project administration, Resources, Supervision, Validation, Writing – review & editing. MM: Data curation, Investigation, Methodology, Writing – review & editing. RV-C: Conceptualization, Data curation, Formal analysis, Funding acquisition, Investigation, Methodology, Project administration, Resources, Supervision, Writing – review & editing. PM: Conceptualization, Funding acquisition, Investigation, Methodology, Project administration, Resources, Supervision, Writing – review & editing. JF: Investigation, Methodology, Project administration, Supervision, Writing – review & editing. MS: Conceptualization, Data curation, Formal analysis, Funding acquisition, Investigation, Methodology, Project administration, Resources, Software, Supervision, Validation, Visualization, Writing – review & editing.

## Footnotes

^1^ https://www.bioinformatics.babraham.ac.uk/projects/fastqc/

^2^ https://gist.github.com/gpertea/b83f1b32435e166afa92a2d388527f4b

^3^ https://cran.r-project.org/package=pheatmap

^4^ https://github.com/kevinblighe/PCAtools
